# Effects of Body Mass Index and Pay-for-Performance Program on Risk of Death in Patients with Type 2 Diabetes: A Nationwide Cohort Study

**DOI:** 10.3390/ijerph18094648

**Published:** 2021-04-27

**Authors:** Hsiu-Ling Huang, Chuan-Yu Kung, Shun-Mu Wang, Pei-Tseng Kung, Yen-Hsiung Lin, Li-Ting Chiu, Wen-Chen Tsai

**Affiliations:** 1 Department of Senior Services Industry Management, Minghsin University of Science and Technology, Hsinchu County 304, Taiwan; hmling88@gmail.com (H.-L.H.); weal1993@yahoo.com.tw (S.-M.W.); 2 Department of Nursing, Hengchun Tourism Hospital, Ministry of Health and Welfare, Pingtung County 94641, Taiwan; kama@pnhb.mohw.gov.tw; 3 Department of Healthcare Administration, Asia University, Taichung City 41354, Taiwan; ptkung@asia.edu.tw; 4 Department of Medical Research, China Medical University Hospital, China Medical University, Taichung City 40402, Taiwan; 5 Department of Pediatrics, Hengchun Tourism Hospital, Ministry of Health and Welfare, Pingtung County 94641, Taiwan; yhs_lin@yahoo.com.tw; 6 Department of Health Services Administration, China Medical University, Taichung 40402, Taiwan; litingchiu933@gmail.com

**Keywords:** body mass index, diabetic, pay-for-performance, death risk

## Abstract

Background: The diabetes patients enrolled in the pay-for-performance (P4P) program demonstrate reduced risk of death. Body mass index (BMI) is a risk factor of all-cause death. This study investigates the effects of BMI and P4P on the risk of death in type 2 diabetes patients. Methods: This is a retrospective cohort study. The study population includes the 3-wave National Health Interview Survey in Taiwan. A total of 6354 patients with diabetes aged ≥ 20 years were enrolled and followed up until the end of 2014. Results: The highest mortality rate per 1000 person-years was 61.05 in the underweight patients with diabetes. A lower crude death rate was observed in the P4P participants than non-P4P participants. The risk of death was 1.86 times higher in the underweight patients with diabetes than that in the normal weight group (95% CI: 1.37–2.53) and was lower in the P4P participants, as compared to the non-participants (HR: 0.55, 95% CI: 0.44–0.69). The most significant effect of joining the P4P program in reducing death risk was found in the underweight patients with diabetes (HR: 0.11, 95% CI: 0.04–0.38), followed by the obesity group (HR: 0.30, 95% CI: 0.17–0.52). Conclusions: Different effects of joining the P4P program on reducing death risk were observed in the underweight and obesity groups. We strongly recommend that patients with diabetes and without healthy BMIs participate in the P4P program.

## 1. Introduction

Diabetes Mellitus (DM) is one of the most important chronic diseases in the world. According to the International Diabetes Federation (IDF) [1], in 2019, there were 463 million people with diabetes, and that number is expected to rise to 578 million by 2030. Previous studies have shown that the life expectancy for a 30-year-old person with diabetes is 11 years shorter than an individual without diabetes [2]; while a 50-year-old male with diabetes can expect to live 3.2 years less than a non-diabetic male. Additionally, women with diabetes have a 3.1-year-shorter life expectancy than women without the disease [3]. Therefore, early intervention is of great importance to effectively control risk factors and extend life expectancy.

Obesity is one of the most significant risk factors for various chronic diseases, including diabetes. Previous studies have revealed the higher the body mass index (BMI, kg/m^2^) of an individual, the more likely they are to have metabolic disorders such as diabetes [4,5]. The prevalence of overweight and obesity in adults in Taiwan is 20.8% and 27.1%, respectively, and all-cause mortality has been shown to increase with increases in BMI in obese people [6]. A similar phenomenon is also found in western populations; in a study of 1.46 million white adults, Berrington et al. [7] reported an increased risk of all-cause death in overweight, obese, and underweight populations. Compher et al. [8] showed that a higher risk of death occurs in the underweight (BMI < 18.5) people, as compared to those with a normal BMI (18.5 < BMI < 24.9), both in Asian and in Western populations. Bhaskaran et al. [9] also suggested that higher and lower BMI independently increases mortality risk.

In a previous study, Zaccardi et al. [10], including 414,587 participants with type 2 diabetes, reported that BMI was associated with all-cause mortality and had the lowest risk in the overweight group in both sexes. Another study also showed an obesity paradox phenomenon for patients with type 2 diabetes concerning all-cause and cardiovascular mortality [11]. A similar found was reported by Edqvist et al. [12]; the study findings suggest that overweight (BMI 25 to < 30 kg/m^2^) patients with type 2 diabetes had low excess mortality risk compared to control subjects. In contrast, the risk in those with BMI ≥ 40 kg/m^2^ was substantially increased.

Since 2001, to effectively improve the clinical condition of patients with diabetes, Taiwan has implemented the “Improvement Program of National Health Insurance Payment for Diabetic Medical Treatment” [13], which is also called the “Pay-For-performance, P4P” program, under which, payment is dependent on the performance of healthcare [14,15,16]. The P4P program aims to supply the integration of diabetic care services, enhancing management of disease and decreasing the loss of life and expenditure.

Previous studies have demonstrated reduced hospitalization and emergency department utilization in patients participating in the P4P program, in addition to shorter hospital stays, reduced medical expenses, better quality of care for patients with diabetes, and the extension of quality-adjusted life-years (QALYs) [17]. Chen et al. [14] reported a decrease in risk of death in patients with diabetes in the P4P group by 0.76-fold compared to those in the non-P4P group (95% CI: 0.64–0.92). On the other hand, Mendelson et al. [15] reported improved process-of-care outcomes over the short term (2 to 3 years) for patients in the P4P program, but long-term effects were limited.

In sum, previous studies showed patients with diabetes under the P4P program have a reduced risk of death [14] with extended QALYs [17]. Various studies have examined the relationship between BMI and mortality in patients with type 2 diabetes [6,7,8]. However, it is unclear whether the effects of P4P on the risk of death are different in patients with diabetes when the patients have different BMIs. Therefore, we aim to explore the impact of the interaction effect between P4P participation status and BMI on the risk of death in patients with diabetes. Our results provide an essential reference for improving the effectiveness of the P4P intervention program for patients with diabetes.

## 2. Materials and Methods

### 2.1. Data Sources and Participants

In this retrospective cohort study, the height and weight data of a total of 60,603 people were used to calculate BMI from the “National Health Interview Survey in Taiwan” in 2001, 2005, and 2009, provided by National Health Promotion Admission, Ministry of Health and Welfare. We extended the wash-out period to 1 January 2000, for our participants in this study. Then, by linking the people to the National Health Insurance Research Database (NHIRD) from 2000 to 2014, screening for patients with type 2 diabetes and ≥20 years old, ruling out those who were pregnant or with other types of diabetes at that time, a total of 6354 patients met the specified criteria. All participants survived until at least 31 December 2014.

The data in this study have been de-identified, personal identity was fully protected, and the study was approved by the Research Ethics Committee, China Medical University (IRB No: CMUH 103-REC3-109).

The National Health Interview Survey in Taiwan is a nationally representative survey of population health and long-term trends. The survey has been conducted every four years to collect policy-relevant information for priority setting and objective establishment. The contents of the questionnaire are personal characteristics, health status, knowledge of disease prevention, medical and preventive care utilization, health behaviors, self-awareness of health status, occupation and economic status [18].

National Health Insurance (NHI) in Taiwan was launched in March 1995. By the end of 2018, more than 23 million people had been insured, and 99.82% of the population is covered by NHI [19]. NHI is a compulsory enrolment program for all citizens and legal residents and provides health care insurance for all residents living in Taiwan. NHI covers all necessary medical expenses, including prescriptions, treatments, operations, and investigations in outpatient visits, inpatient and emergency systems; the database contains basic demographic and medical information, including treatment of diabetes [20,21]. Health insurance information has become representative empirical data in medical and health research, and the results are often used as references for medical and health policy [22,23,24].

In Taiwan, the DM-P4P program aims to motivate medical institutions to participate in the care of patients with diabetes and establish a quality monitoring mechanism and notification system. Financial incentives, specific quality indicators, and clinical guidelines [14] are applied to promote integration within a medical institution, establish continuous medical care, and formulate bundled payment plans. Before the NHI’s approval, medical institutions should organize a medical team, including physicians, nurse practitioners, nutritionists, and health education professionals. There is no primary care gatekeeping in Taiwan and no complete referral system, and patients are free to seek health care based on their discretion. Health care institutions with physicians can voluntarily apply to participate in the NHI P4P program. These certified physicians can enrol patients individually into the program, and patients are free to participate in the program. To encourage medical institutions to join the P4P program, patients who have been diagnosed with diabetes and have paid more than two visits to the same medical institution within 90 days can be enrolled in the program. The medical team should adhere to the clinical guidelines: HbA1c < 7%, blood pressure < 130/80 mmHg, and low-density lipoprotein cholesterol < 100 mg/dl or total cholesterol < 160 mg/dl [25,26]. The medical team in the P4P program should provide appropriate medical care, report the case management fees regularly, and register related quality information as requested. When the medical institutions achieve the expected goals or improve care results, they can apply for the NHI management care fee.

The DM-P4P program provides financial rewards to medical institutions to increase medical measure improvements (e.g., HbA1c, blood pressure, and low-density lipoprotein cholesterol, total cholesterol, the incidence of stroke, myocardial infarction, heart failure, and foot ulcers) and achieve optimal in process-of-care outcomes (e.g., physician visits, specific examinations) [25,27,28] and health outcomes (e.g., complications, survival) [15,29] in patients with diabetes.

### 2.2. Variables Description

The variables in this study are BMI (underweight, normal, overweight, mild obesity and obesity), the diabetic patient’s participation in the P4P program or not, survival or not, personal characteristics (gender, age, education, and marital status), environmental factors (degree of urbanization), social and economic status (monthly salary), health status (Charlson comorbidity index (CCI), diabetes complication severity index (DCSI)), health behavior (smoking, drinking, weekly energy expenditure in exercise) and the level of primary healthcare organizations (medical center, regional hospital, district hospital, community clinic).

The WHO has recommended classifications for bodyweight since the cut-off points for determining overweight and obesity in Southeast Asian adults are lower than those of the world average. Different Asian countries have the cut-off points for overweight and obesity tailored to their populations. In this study, we adopt the BMI cut-off points recommended by Health Promotion Administration, Ministry of Health and Welfare Taiwan, i.e., underweight (BMI < 18.5), normal BMI (18.5 ≤ BMI < 24), overweight (24 ≤ BMI < 27), mild obesity (27 ≤ BMI < 30), and obesity (BMI ≥ 30) [30].

Using NHIRD, we identified patients diagnosed with diabetes according to the International Classification of Diseases, Ninth Revision, Clinical Modification (ICD-9-CM code). The individuals who had at least three outpatient visits or one hospitalization with a principal or secondary diagnosis of diabetes (ICD-9-CM: 250) within consecutive 365 days were defined as the study population [31]. The patients diagnosed with type I diabetes (ICD-9-CM: 6488), gestational diabetes (ICD-9-CM: 7751), neonatal diabetes mellitus (ICD-9-CM: 7751), or impaired glucose tolerance (ICD-9-CM: 6488) were excluded.

The data in this study are linked to the cause of death data. The individual was defined as dead only through registration in the database during the observation period; otherwise, they were classed as having survived. The patients with diabetes enrolled in the P4P program are defined as the P14xx internal code in the National Health Insurance Database [32].

The urbanization degree of residential areas for the participants was divided into 7 levels; the highest degree of urbanization was defined as level 1, and the lowest as level 7 [33]. The severity of comorbidity was calculated by Deyo’s Charlson comorbidity index (CCI) and was given different weights depending on the severity [34], defined as 0, 1, 2, and ≥ 3. According to Young et al. [35], the Diabetes Complication Severity Index (DCSI) is classified into seven types of complications, including retinopathy, nephropathy, cerebrovascular, cardiovascular, peripheral vascular disease, and metabolic. It is given different weights depending on the severity, as 0, 1, 2, and ≥ 3.

In terms of health behavior, weekly energy expenditure through exercise was calculated according to Wen et al. [36]. In brief, the correlation between the type of exercise, self-aware breathing status, and metabolic equivalent task (MET) was proposed based on the “National Health Interview Survey in Taiwan” in 2007. One MET is defined as 1 kcal/kg/hour, and the oxygen uptake when quietly sitting is approximately 3.5 mL/kg/min. Weekly energy expenditure equals MET * Frequency of exercise over the past two weeks (times) * Each exercise duration (hour) * Bodyweight (Kg) * 7/14. There is a unique MET for each type of exercise according to the breath status. Weekly energy expenditure for specific exercise (kcal) is calculated via MET. It is divided into no exercise, < 500 kcal/week, and ≥ 500 kcal/week.

The level of primary healthcare organizations is defined as the most frequent medical organization visited because of diabetes. If the frequency of two organizations were the same, the most recent organization met was chosen. The organizations were classified as a medical center, regional hospital, district hospital, and community clinic.

### 2.3. Statistical Analysis

SAS 9.4 (SAS Institute, Cary, NC, USA) software was applied for the analysis, and significance was defined as *p*-values < 0.05. Descriptive statistics were used to describe the study participant demographics and P4P participation status of patients with diabetes in each variable; univariate Poisson regression was used to examine the difference in mortality rates between groups within a variable; Cox proportional hazard model was applied to explore the risk of death of patients with diabetes with different BMIs, and interactive effects of BMI and P4P on the risk of death, i.e., whether there are differential effects of P4P on the risk of death of DM patients based on different BMIs. The study conducted a stratified analysis of BMI level. The Cox proportional hazard model was used to examine the effect of the P4P program on reducing the death risk in each BMI group.

## 3. Results

### 3.1. Essential Characteristics of Patients with Type 2 Diabetes and Mortality Rate per 1000 Person-Years

As shown in Table 1, 6354 patients with diabetes met the criteria of enrolment. Among them, 1946 were enrolled in the P4P program (30.63%), and 4408 (69.37%) were not. The participation distribution was significantly different (*p* < 0.05) between P4P and non-P4P groups in variables including BMI level, age, education level, marital status, urbanization of residence area, monthly salary, CCI, DCSI, drinking, weekly energy expenditure during exercise, and primary healthcare organization. Almost all *p*-values were significant except for gender and smoking.

As shown in Table 2, there were 1162 deaths (18.29%) in the follow-up period, with an average follow-up of 8.06 ± 4.51 years, and the overall annual mortality rate with an incidence of per 1000 person-years was 22.68. Comparing the mortality rates, the highest mortality rate per 1000 person-years of 61.05 was in underweight DM patients (BMI < 18.5); there was a lower mortality in patients with diabetes after participation in the P4P program when compared to before participation (12.17 vs. 28.74 cases per 1000 person-years, respectively); a lower mortality was found in female DM patients than in males (18.82 vs. 26.45 cases per 1000 person-years, respectively). The older the DM patients, the higher the mortality rate, i.e., it reached 81.58 per 1000 person-years when DM patients were older than 75 years old. Lower mortality rates were found in patients with higher education levels; higher mortality rates were found in patients with higher CCI and DSCI. As for health behavior, a lower mortality rate was found in DM patients who did not smoke, drank once per week, and had a higher weekly energy expenditure (kcal).

### 3.2. Comparison of Mortality Rate per 1000 Person-Years in DM Patients with Different BMIs in P4P or Non-P4P Program

Univariate Poisson regression was employed to compare the mortality rate per 1000 person-years in DM patients before and after joining P4P (Table 3). It revealed a significantly lower mortality rate in each variable for DM patients in the P4P group (*p* < 0.05). Further exploration of the P4P impact on the annual mortality rate, with an incidence of per 1000 person-years in DM patients with different BMIs, indicates that irrespective of the BMI level, there were significantly lower mortality rates (*p* < 0.05) in P4P DM patients as compared to non-P4P DM patients with the same BMI (Table 3). The highest mortality rate was found in underweight patients with diabetes (BMI < 18.5) in both P4P and non-P4P groups (P4P vs. non-P4P = 27.98 vs. 74.62 cases per 1000 person-years).

### 3.3. The Effects of BMI and P4P on the Risk of Death in Patients with Type 2 Diabetes and Related Factors

To understand whether the risk of death in patients with diabetes is affected by BMI and P4P, we used the Cox proportional hazard model to investigate the risk of death and related factors and the interaction between P4P and BMI. As shown in Table 4, a 1.86-fold increase was found in underweight (BMI < 18.5) DM patients as compared to those with a normal BMI (18.5 ≤ BMI < 24) (95% CI: 1.37–2.53, *p* < 0.05). A lower risk of death was detected in DM patients in the P4P group compared to those not participating in the P4P program (Adj. HR: 0.55, 95% CI: 0.44–0.69, *p* < 0.05). Differential interaction between BMI and P4P was found when the BMI level was ≥ 24; the P4P program has a different effect on reducing the risk of death in DM patients with different BMIs. This study also found a lower risk of death (*p* < 0.05) in female DM patients and those who had higher weekly energy expenditures. However, a higher mortality rate was found in older patients, higher CCI, and DCSI scores (*p* < 0.05).

### 3.4. Comparing the Effects of P4P on Death Risk in Patients with Type 2 Diabetes at Different BMI Levels by Stratified Analysis

**Figure 1:**
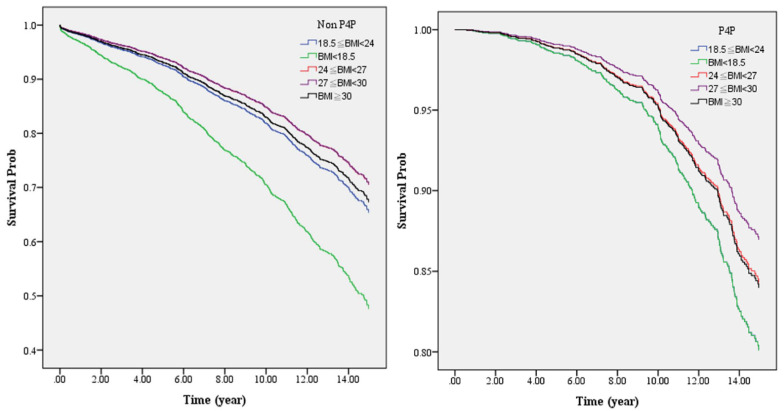
Comparing the effects of P4P and non-P4P on the death risk in patients with different BMIs via Cox proportional hazard model (after controlling for sex, age, education level, marital status, urbanization of residence area, monthly salary, CCI, DCSI, smoking, drinking, weekly energy expenditure through exercise, and primary healthcare organizations).

**Figure 2:**
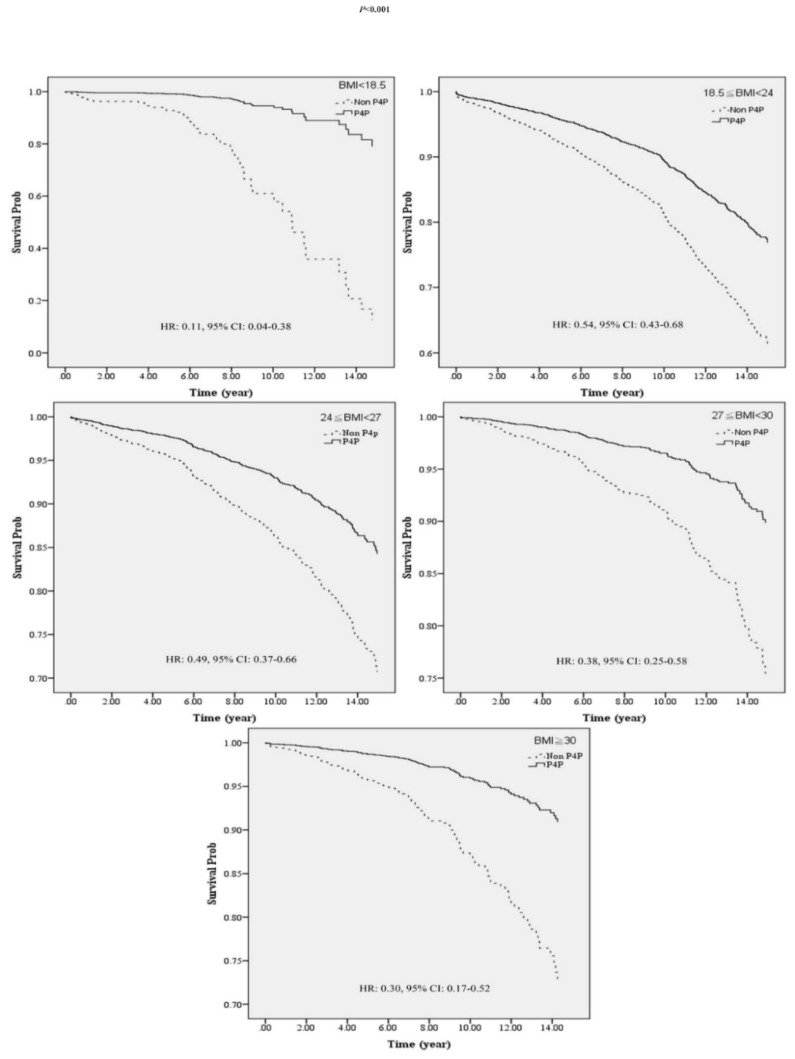
Stratified analysis was conducted to compare the differences in reducing the risk of death between P4P and non-P4P groups by BMI category.

Further investigation of the effects of P4P on the death risk in patients with diabetes with different BMIs via the Cox proportional hazard model revealed, as shown in Figure 1, that irrespective of participation in P4P or not, the highest risk of death was noticed in underweight patients with diabetes (BMI < 18.5). After patients were enrolled in the P4P program, the study showed a significantly greater decrease in the risk of death in underweight P4P patients, resulting in the mortality being closer to patients with other BMIs in the same group (Figure 1). This study conducted a stratified analysis and divided the BMIs of the patients with diabetes into five levels (Table 5, Figure 2). After controlling for other variables, a significantly lower risk of death was found in the P4P group as compared to non-P4P participants with the same BMI (*p* < 0.05); among them, the most significant effect of joining the P4P program on reducing the risk of death was found in the underweight patients with diabetes (BMI < 18.5, Adj. HR: 0.11, 95% CI: 0.04–0.38), followed by the obesity group (BMI ≥ 30, Adj. HR: 0.30, 95% CI: 0.17–0.52).

## 4. Discussion

To our knowledge, this is the first study to investigate the difference in the risk of death among diabetic patients with different BMIs by participating in the P4P program. The results reveal a significantly reduced mortality rate per 1000 person-years for patients with diabetes in the P4P group compared to those in the non-P4P group (*p* < 0.05, Table 3), with the mortality rate of P4P and non-P4P groups being 12.17 vs. 28.74, respectively. The risk of death was reduced to 0.55-fold that of the non-P4P participants (95% CI: 0.44–0.69, *p* < 0.05, Table 4). Similar results were reported by Chen et al. [14], namely, that the mortality rate of the P4P group was reduced by 0.76-fold compared to that of the non-P4P group (*p* < 0.05), and a significantly longer survival time was found in the P4P group. Pan et al. [37] also reported higher physician continuity and a lower risk of death in patients with diabetes who participated in the P4P program. Lee et al. [27] showed that patients in the P4P program received significantly more diabetes-specific exams and tests than patients who were not enrolled. The patients in the P4P group had an average of two more physician visits for diabetes than those in the comparison group. Wu et al. [38] reported that participation in the P4P program might have reduced the risk of ER infection events and resulted in lower infection-related deaths in patients with type 2 diabetes. Despite the previous demonstration of the benefits of P4P participation for patients with diabetes in reducing the risk of death [14,37], there has been no report including BMI as a variable. Here, we analyzed and revealed the differential effects of reducing the risk of death via participation in P4P in patients with diabetes with different BMIs (Table 5 and Figure 2).

When comparing the P4P participants with non-participants, we found a higher mortality rate in underweight DM patients than in patients with other BMI levels in both the P4P and non-P4P group (Table 3). A previous study found a nonlinear relationship between BMI and all-cause mortality in patients with type 2 diabetes [10], which showed that when the BMI of patients with diabetes is too low, the risk of death is much higher, irrespective of gender. Tobias et al. [39] also reported a higher risk of all-cause of death in underweight patients with diabetes (BMI of 18.5–22.4), as compared to the control group (BMI of 22.5–24.9). Similar results were reported by Vestberg et al. [40], namely, that a five-fold increase in the risk of coronary artery diseases (HR: 5.0, 95% CI: 1.5–16.9) and a 5.4-fold increase in the risk of all-cause mortality (HR: 5.4, 95% CI: 3.1–9.6) was found in underweight type I patients with diabetes (BMI < 18.5) as compared to the control group (BMI of 18.5–25). Compher et al. [8] also reported increased mortality in both Western and Asian underweight patients (BMI < 18.5) compared to the normal group (BMI of 18.5–24.9). However, the most significant effects of participating in the P4P program on reducing the risk of death was found in underweight patients with diabetes (BMI < 18.5) (HR = 0.11, 95% CI: 0.04–0.38, Table 5), which is a significant finding for improving the effectiveness of P4P policy.

It is also noteworthy that overweight patients with diabetes (24 ≤ BMI < 27) have a lower risk of death than patients with normal BMIs (HR: 0.83, *p* < 0.05, Table 4); however, no significant difference (*p* > 0.05) in the risk of death was found when the patients were mildly obese or above (BMI ≥ 27). Similarly, Liu et al. [41] reported a lower risk of all-cause of death in overweight and obese patients with diabetes when compared to those with normal BMI (RR: 0.81 vs. 0.72); Huang et al. [42] showed lower hip fracture risks in patients with diabetes who were overweight or obese (HR: 0.49 or 0.42, *p* < 0.05). Further, we also examined the interaction relationship between P4P participation status and BMI level in relation to the risk of death. As shown in Table 4, we found that obese (BMI ≥ 30) patients with type 2 diabetes who joined the P4P program had the lowest excess mortality risk compared to those with normal BMIs, followed by the mildly obese group (27 ≤ BMI < 30). Therefore, we suggest that patients with type 2 diabetes with higher BMIs should participate in the P4P program, which could result in a lower mortality rate. Despite the fact that a significantly decreased risk of death was found in overweight patients with diabetes (24 ≤ BMI < 27, *p* < 0.05), overweight is a significant public health problem. Specifically, being overweight could lead to hypertension, coronary artery disease, and cerebrovascular disease; therefore, it is recommended that patients with diabetes should maintain a normal BMI.

In this study, the Cox proportional hazard model was employed to clarify the interaction and benefit BMI and P4P on the risk of death of patients with diabetes. The results showed interactions between BMI and P4P on the risk of death when the BMI was ≥ 24 (*p* < 0.05, Table 4). In the stratified analysis, as shown in Table 5, participation in P4P reduced the death risk in patients with diabetes with different BMIs (*p* < 0.05). The most significant benefit was found in underweight patients with diabetes (BMI < 18.5, Adj. HR: 0.11), followed by the obesity group (BMI ≥ 30, Adj. HR: 0.30). P4P is a form of medical care management, which integrates healthcare by physicians, nurse practitioners, nutritionists, and health education professionals for diet education, weight control, and regular follow-up for patients with diabetes. Continuous and comprehensive disease management is provided in the program to improve the accessibility of the healthcare system and effectively control the disease progression [13]. Based on our findings of a more pronounced effect on reducing the risk of death in underweight or obese patients with diabetes participating in the P4P program, we highly recommend that underweight and obese patients with diabetes participate in the P4P program and control risk factors as early as possible.

## 5. Limitations

This is a retrospective study and was based on the health insurance medical claim database; patient information such as blood glucose control, HbA1c, the level of C-reactive protein, cause of death, and compliance to diabetic prescriptions was unavailable. Therefore, no clinical data were obtained to verify the accuracy of diabetes in the NHIRD, where the ICD-9-CM codes were applied. To optimize the accuracy of diabetes diagnosis and compensate for this limitation, this study defined diabetes as patients who had at least three outpatient visits or one hospitalization with the primary or secondary diagnosis of diabetes (ICD-9-CM: 250) within the consecutive 365 days [31].

## 6. Conclusions

In this study, we found (1) the mortality rate per 1000 person-years of patients with diabetes in the P4P group was lower than that of P4P non-participants (*p* < 0.05); (2) the death risk of diabetic P4P participants was 0.55-fold lower than that of P4P non-participants; (3) there were differential effects of BMIs on the risk reduction by participation in the P4P program when patients were overweight (BMI ≥ 24, *p* < 0.05); (4) slightly overweight patients with diabetes (24 ≤ BMI < 27) had lower risks of death (Adj. HR: 0.83, *p* < 0.05); (5) P4P reduced the risk of death in patients with diabetes with different BMIs, with the most significant effect found in underweight DM patients (BMI < 18.5), followed by the obese group (BMI ≥ 30).

Based on the findings, we recommend that patients with diabetes participate in the P4P program, especially underweight and obese patients; Additionally, patients with diabetes should maintain a healthy BMI and avoid becoming underweight, reducing the risk of death effectively. The results of this study can be applied as references for planning the health promotion interventions in patients with diabetes.

## Figures and Tables

**Table 1:** The demographic characteristics of patients with type 2 diabetes.

Variable	Total	%	non-P4P		P4P		*p*-Value
			*n*	%	*n*	%	
**Total**	6354	100.00	4408	69.37	1946	30.63	
**BMI**						<0.001	
BMI < 18.5	133	2.09	108	2.45	25	1.28	
18.5 ≤ BMI < 24	2064	32.48	1483	33.64	581	29.86	
24 ≤ BMI < 27	2064	32.48	1421	32.24	643	33.04	
27 ≤ BMI < 30	1240	19.52	834	18.92	406	20.86	
BMI ≥ 30	853	13.42	562	12.75	291	14.95	
**P4P**							
No	4408	69.37	-	-	-	-	
Yes	1946	30.63	-	-	-	-	
**Survival**						<0.001	
No	5192	81.71	3474	78.81	1718	88.28	
Yes	1162	18.29	934	21.19	228	11.72	
**Sex**						0.236	
Male	3328	52.38	2331	52.88	997	51.23	
Female	3026	47.62	2077	47.12	949	48.77	
**Age**						<0.001	
20–44	1020	16.05	684	15.52	336	17.27	
45–54	1619	25.48	1043	23.66	576	29.60	
55–64	1760	27.70	1163	26.38	597	30.68	
65–74	1228	19.33	901	20.44	327	16.80	
≥75	727	11.44	617	14.00	110	5.65	
**Education Level**						0.041	
None or Literate	917	14.43	673	15.27	244	12.54	
Elementary	2299	36.18	1561	35.41	738	37.92	
Junior	998	15.71	684	15.52	314	16.14	
Senior	1276	20.08	894	20.28	382	19.63	
College or above	846	13.31	586	13.29	260	13.36	
Missing	18	0.28					
**Marital Status**						0.001	
Married	4715	74.21	3218	73.00	1497	76.93	
Divorced	266	4.19	177	4.02	89	4.57	
Widowed	704	11.08	517	11.73	187	9.61	
Never married	661	10.40	491	11.14	170	8.74	
Missing	8	0.13					
**Urbanization of Residence Area**						0.006	
Level 1	1326	20.87	962	21.82	364	18.71	
Level 2	1929	30.36	1309	29.70	620	31.86	
Level 3	940	14.79	658	14.93	282	14.49	
Level 5	1237	19.47	830	18.83	407	20.91	
Level 4	199	3.13	154	3.49	45	2.31	
Level 6	420	6.61	284	6.44	136	6.99	
Level 7	303	4.77	211	4.79	92	4.73	
**Monthly Salary (NTD)**						0.042	
≤17,280	1403	22.08	1018	23.09	385	19.78	
17,281–22,800	2879	45.31	1979	44.90	900	46.25	
22,801–28,800	417	6.56	273	6.19	144	7.40	
28,801–36,300	502	7.90	339	7.69	163	8.38	
36,301–45,800	546	8.59	373	8.46	173	8.89	
≥45,801	607	9.55	426	9.66	181	9.30	
**CCI**						0.013	
0	3550	55.87	2425	55.01	1125	57.81	
1	1354	21.31	969	21.98	385	19.78	
2	819	12.89	552	12.52	267	13.72	
≥3	631	9.93	462	10.48	169	8.68	
**DCSI**						<0.001	
0	4942	77.78	3458	78.45	1484	76.26	
1	706	11.11	420	9.53	286	14.70	
2	547	8.61	409	9.28	138	7.09	
≥3	159	2.50	121	2.75	38	1.95	
**Smoking**						0.211	
Never	4526	71.23	3110	70.55	1416	72.76	
Everyday	1525	24.00	1083	24.57	442	22.71	
Quit	297	4.67	210	4.76	87	4.47	
Missing	6	0.09					
**Drinking**						0.013	
Never	4618	72.68	3216	72.96	1402	72.05	
Once a week	880	13.85	577	13.09	303	15.57	
Almost every day	843	13.27	606	13.75	237	12.18	
Missing	13	0.20					
**Weekly Energy Expenditure in Exercise**						0.044	
No exercise	2997	47.17	2123	48.16	874	44.91	
<500 kcal	1193	18.78	819	18.58	374	19.22	
≥500 kcal	2156	33.93	1459	33.10	697	35.82	
Missing	8	0.13					
**Primary Health Care Organizations**						<0.001	
Medical center	1242	19.55	909	20.62	333	17.11	
Regional hospital	1946	30.63	1223	27.75	723	37.15	
District hospital	1274	20.05	887	20.12	387	19.89	
Community clinic	1892	29.78	1389	31.51	503	25.85	

BMI: body mass index; P4P: pay-for-performance; CCI: Charlson Comorbidity Index; DCSI: diabetic complication severity index; NTD: New Taiwan Dollar; NTD 30 = USD 1. Urbanization of residence area (overall 7 levels; level 1 was the most urbanized); *p*-value: chi-square test.

**Table 2:** The mortality rate of diabetic per 1000 person-years.

Variable	Total	Death (N)	Total Person-Years	Incidence/1000 Person-Years	*p*-Value ^#^
**Total**	6354	1162	51,233.56	22.68	-
**BMI**					
BMI < 18.5	133	60	982.83	61.05	<0.001
18.5 ≤ BMI < 24	2064	512	17,090.74	29.96	-
24 ≤ BMI < 27	2064	319	16,610.69	19.20	<0.001
27 ≤ BMI < 30	1240	159	9947.51	15.98	<0.001
BMI ≥ 30	853	112	6601.78	16.97	<0.001
**P4P**					
No	4408	934	32,503.77	28.74	-
Yes	1946	228	18,729.79	12.17	<0.001
**Sex**					
Male	3328	686	25,940.12	26.45	-
Female	3026	476	25,293.43	18.82	<0.001
**Age**					
20–44	1020	69	8430.75	8.18	-
45–54	1619	144	13,703.10	10.51	0.088
55–64	1760	230	14,817.12	15.52	<0.001
65–74	1228	362	9906.48	36.54	<0.001
≥75	727	357	4376.11	81.58	<0.001
**Education Level**					
None or Literate	917	313	8001.55	39.12	-
Elementary	2299	496	19,195.10	25.84	<0.001
Junior	998	126	7814.75	16.12	<0.001
Senior	1276	139	9586.19	14.50	<0.001
College or above	846	78	6523.90	11.96	<0.001
Missing	18				
**Marital Status**					
Married	4715	758	38,788.19	19.54	-
Divorced	266	40	1946.93	20.55	0.758
Widowed	704	203	5935.65	34.20	<0.001
Never married	661	159	4509.52	35.26	<0.001
Missing	8				
**Urbanization of Residence Area**					
Level 1	1326	221	10,999.86	20.09	-
Level 2	1929	309	15,803.21	19.55	0.004
Level 3	940	171	7619.56	22.44	0.001
Level 5	1237	253	9580.61	26.41	0.047
Level 4	199	43	1495.23	28.76	0.384
Level 6	420	92	3272.64	28.11	0.874
Level 7	303	73	2462.44	29.65	0.735
**Monthly Salary (NTD)**					
≤17,280	1403	340	11,247.83	30.23	-
17,281–22,800	2879	572	24,138.51	23.70	<0.001
22,801–28,800	417	45	3301.53	13.63	<0.001
28,801–36,300	502	58	3768.27	15.39	<0.001
36,301–45,800	546	67	4045.07	16.56	<0.001
≥45,801	607	80	4732.34	16.90	<0.001
**CCI**					
0	3550	406	28,926.31	14.04	-
1	1354	266	10,878.79	24.45	<0.001
2	819	200	6834.13	29.26	<0.001
≥3	631	290	4594.32	63.12	<0.001
**DCSI**					
0	4942	760	39,569.14	19.21	-
1	706	135	6396.50	21.11	0.313
2	547	191	4029.83	47.40	<0.001
≥3	159	76	1238.08	61.39	<0.001
**Smoking**					
Never	4526	747	37,833.21	19.74	-
Everyday	1525	303	10,946.25	27.68	<0.001
Quit	297	111	2426.88	45.74	<0.001
Missing	6				
**Drinking**					
Never	4618	932	38,379.95	24.28	-
Once a week	880	104	6776.01	15.35	<0.001
Almost everyday	843	125	5979.73	20.90	0.116
Missing	13				
**Weekly Energy Expenditure in Exercise**					
No exercise	2997	615	22,852.01	26.91	-
<500 kcal	1193	199	9958.23	19.98	<0.001
≥500 kcal	2156	346	18,376.51	18.83	<0.001
Missing	8				
**Primary Healthcare Organizations**					
Medical center	1242	280	10,397.39	26.93	-
Regional hospital	1946	408	16,027.29	25.46	0.469
District hospital	1274	265	10,196.84	25.99	0.678
Community clinic	1892	209	14,612.03	14.30	<0.001

^#^ univariate Poisson regression BMI: body mass index; P4P: pay-for-performance; CCI: Charlson Comorbidity Index; DCSI: diabetic complication severity index; NTD: New Taiwan Dollar; NTD 30 = USD 1. Urbanization of residence area (overall 7 levels; level 1 was the most urbanized); *p*-value less than 0.05 was considered statistically significant.

**Table 3:** Comparison of mortality rate per 1000 person-years in DM patients with different BMIs after participation in P4P program.

	Non-P4P				P4P				
Variable	Total	Death (N)	Total Person-Years	Incidence/ 1000 Person-Years	Total	Death (N)	Total Person-Years	Incidence/ 1000 Person-Years	*p*-Value ^#^
**Total**	4408	934	32,503.77	28.74	1946	228	18,729.79	12.17	<0.001
**BMI**									
BMI < 18.5	108	52	696.91	74.62	25	8	285.92	27.98	0.010
18.5 ≤ BMI < 24	1483	411	11,189.29	36.73	581	101	5901.45	17.11	<0.001
24 ≤ BMI < 27	1421	252	10,435.89	24.15	643	67	6174.80	10.85	<0.001
27 ≤ BMI < 30	834	126	6148.21	20.49	406	33	3799.30	8.69	<0.001
BMI ≥ 30	562	93	4033.47	23.06	291	19	2568.32	7.40	<0.001
**Sex**									
Male	2331	556	16,693.68	33.31	997	130	9246.44	14.06	<0.001
Female	2077	378	15,810.08	23.91	949	98	9483.35	10.33	<0.001
**Age**									
20–44	684	57	5385.08	10.58	336	12	3045.67	3.94	0.002
45–54	1043	111	8048.34	13.79	576	33	5654.76	5.84	<0.001
55–64	1163	155	8901.86	17.41	597	75	5915.26	12.68	0.024
65–74	901	286	6646.42	43.03	327	76	3260.07	23.31	<0.001
≥75	617	325	3522.07	92.28	110	32	854.04	37.47	<0.001
**CCI**									
0	2425	317	18,291.86	17.33	1125	89	10,634.45	8.37	<0.001
1	969	219	7157.38	30.60	385	47	3721.41	12.63	<0.001
2	552	153	4150.19	36.87	267	47	2683.95	17.51	<0.001
≥3	462	245	2904.34	84.36	169	45	1689.98	26.63	<0.001
**DCSI**									
0	3458	600	25,386.28	23.63	1484	160	14,182.85	11.28	<0.001
1	420	100	3513.65	28.46	286	35	2882.86	12.14	<0.001
2	409	168	2772.19	60.60	138	23	1257.64	18.29	<0.001
≥3	121	66	831.65	79.36	38	10	406.44	24.60	0.001
**Weekly Energy Expenditure in Exercise**									
No exercise	2123	519	14,926.12	34.77	874	96	7925.88	12.11	<0.001
<500 kcal	819	152	6212.30	24.47	374	47	3745.92	12.55	<0.001
≥500 kcal	1459	261	11,320.28	23.06	697	85	7056.22	12.05	<0.001
missing	7				1				

^#^ univariate Poisson regression. BMI: body mass index; P4P: pay-for-performance; CCI: Charlson Comorbidity Index; DCSI: diabetic complication severity index. NTD: New Taiwan Dollar; NTD 30 = USD 1. Urbanization of residence area (overall 7 levels; level 1 was the most urbanized). *p*-value less than 0.05 was considered statistically significant.

**Table 4:** The effects of BMI and P4P and related factors on the risk of death in patients with diabetes (results of Cox proportional hazard model).

Variable	Unadjusted Model				Adjusted Model			
	HR	95% CI		*p*-Value	HR	95% CI		*p*-Value
**BMI**								
BMI < 18.5	2.12	1.62	2.77	<0.001	1.86	1.37	2.53	<0.001
18.5 ≤ BMI < 24	1.00	-	-	-	1.00	-	-	-
24 ≤ BMI < 27	0.66	0.57	0.76	<0.001	0.83	0.71	0.97	0.023
27 ≤ BMI < 30	0.54	0.46	0.65	<0.001	0.83	0.68	1.02	0.070
BMI ≥ 30	0.59	0.48	0.73	<0.001	0.95	0.75	1.20	0.668
**P4P**								
No	1.00	-	-	-	1.00	-	-	-
Yes	0.39	0.34	0.45	<0.001	0.55	0.44	0.69	<0.001
P4P*BMI								
P4P*(BMI < 18.5)					0.86	0.41	1.82	0.699
P4P*(18.5 ≤ BMI < 24)					1.00	-	-	-
P4P*(24 ≤ BMI < 27)					0.39	0.30	0.49	<0.001
P4P*(27 ≤ BMI < 30)					0.30	0.21	0.43	<0.001
P4P*(BMI ≥ 30)					0.27	0.17	0.43	<0.001
**Sex**								
Male	1.00	-	-	-	1.00	-	-	-
Female	0.69	0.62	0.78	<0.001	0.67	0.57	0.78	<0.001
**Age**								
20–44	1.00	-	-	-	1.00	-	-	-
45–54	1.26	0.95	1.68	0.110	1.32	0.98	1.78	0.067
55–64	1.84	1.40	2.40	<0.001	1.71	1.28	2.29	<0.001
65–74	4.41	3.41	5.71	<0.001	3.30	2.48	4.38	<0.001
≥75	11.01	8.50	14.26	<0.001	6.32	4.72	8.45	<0.001
**CCI**								
0	1.00	-	-	-	1.00	-	-	-
1	1.74	1.49	2.03	<0.001	1.33	1.13	1.56	<0.001
2	2.02	1.71	2.40	<0.001	1.52	1.27	1.82	<0.001
≥3	4.43	3.81	5.16	<0.001	2.43	2.04	2.88	<0.001
**DCSI**								
0	1.00	-	-	-	1.00	-	-	-
1	1.05	0.87	1.26	0.637	0.84	0.69	1.01	0.065
2	2.48	2.12	2.91	<0.001	1.21	1.02	1.44	0.031
≥3	3.07	2.43	3.89	<0.001	1.20	0.93	1.55	0.167
**Weekly Energy Expenditure in Exercise**								
No exercise	1.00	-	-	-	1.00	-	-	-
<500 kcal	0.72	0.61	0.84	<0.001	0.85	0.73	1.01	0.059
≥500 kcal	0.68	0.60	0.78	<0.001	0.82	0.72	0.95	0.006

Note: The participants of patients with diabetes had an average follow-up of 8.06 ± 4.51 years. BMI: body mass index; P4P: pay-for-performance; CCI: Charlson Comorbidity Index; DCSI: diabetic complication severity index. NTD: New Taiwan Dollar; NTD 30 = USD 1 dollar. Urbanization of residence area (overall 7 levels; level 1 was the most urbanized). *p*-value less than 0.05 was considered statistically significant.

**Table 5:** Comparing the effects of P4P on death risk in patients with diabetes with different BMIs by stratified analysis.

	Non-P4P			P4P			Adj. HR	95% CI		*p*-Value
	N	Death		N	Death		(P4P vs. Non-P4P)			
Variable		*n*	%		*n*	%				
**Total**	4408	934	21.19	1946	228	11.72				
**BMI**										
BMI < 18.5	108	52	48.15	25	8	32.00	0.11	0.04	0.38	<0.001
18.5 ≤ BMI < 24	1483	411	27.71	581	101	17.38	0.54	0.43	0.68	<0.001
24 ≤ BMI < 27	1421	252	17.73	643	67	10.42	0.49	0.37	0.66	<0.001
27 ≤ BMI < 30	834	126	15.11	406	33	8.13	0.38	0.25	0.58	<0.001
BMI ≥ 30	562	93	16.55	291	19	6.53	0.30	0.17	0.52	<0.001

Note: Cox proportional hazards model for each BMI group has been controlled for the relevant variables.

## Data Availability

Data are available from the Science Center, the Ministry of Health and Welfare (MOHW), Taiwan. This study obtained the databases published and managed by the MOHW. All researchers are allowed to use the databases for their interested studies. Before using the databases for research, all studies should get IRB permission.
